# Using Spiritual Connections to Cope With Stress and Anxiety During the COVID-19 Pandemic

**DOI:** 10.3389/fpsyg.2022.915290

**Published:** 2022-07-28

**Authors:** Fahad D. Algahtani, Bandar Alsaif, Ahmed A. Ahmed, Ali A. Almishaal, Sofian T. Obeidat, Rania Fathy Mohamed, Reham Mohammed Kamel, Iram Gul, Sehar un Nisa Hassan

**Affiliations:** ^1^College of Public Health and Health Informatics, University of Ha’il, Ha’il, Saudi Arabia; ^2^Department of Working With Individuals and Families, Faculty of Social Work, Helwan University, Helwan, Egypt; ^3^Department of Social Sciences, College of Arts, University of Ha’il, Ha’il, Saudi Arabia; ^4^College of Applied Medical Sciences, University of Ha’il, Ha’il, Saudi Arabia; ^5^Department of Basic Sciences, Preparatory Year, University of Ha’il, Ha’il, Saudi Arabia; ^6^Department of Psychiatry, Faculty of Medicine, Cairo University, Cairo, Egypt; ^7^Department of Behavioral Sciences, Fatima Jinnah Women University, Rawalpindi, Pakistan

**Keywords:** spirituality, COVID-19, stress, anxiety, psychological, coping

## Abstract

During the initial phases of the COVID-19 pandemic, stress and anxiety were pervasive among the masses due to high morbidity and mortality. Besides the fear of coronavirus was also particularly driven by social media. Many people started to look for faith and spiritual connections to gain comfort. The role of spiritual ties and religious beliefs in relation to coping with pandemic stress has acquired the attention of researchers in some parts of the world. This cross-sectional survey aimed at assessing the intensity of stress and anxiety symptoms experienced by people and how much they were alleviated by employing spiritual connections. The study sample comprises 795 respondents with 52% males and 48% females living in Saudi Arabia. The brief online study questionnaire collected data about background variables, anxiety and stress scale from DASS-21, and items from the WHOQOL (SRBP) instrument assessed the use of spiritual beliefs to cope. Multiple regression models were tested to determine the role of spiritual connections after adjusting demographic variables. Results illustrated that after adjusting for gender and age, participants’ anxiety symptoms decreased by (β = −0.27; *p* = 0.000) units with each unit increase in the use of spiritual connections, and participants’ stress symptoms reduce by (β = −0.36; *p* = 0.000) units with each unit increase in coping with spirituality. Additionally, females’ risk to experience anxiety and stress symptoms was more than males [(β = 0.88; *p* = 0.01) and (β = 0.92; *p* = 0.000)], respectively. An increase in age decreases the likelihood of experiencing anxiety symptoms and stress symptoms by (β = −0.75; *p* = 0.02) and (β = −0.11; *p* = 0.000) units, respectively. Findings support the protective role of spiritual connections despite small beta coefficients. The social and cultural context in Saudi Arabia favors deep-rooted connections with spirituality and faith. Our findings support the fact that the reliance on spiritual connections helped older people to deal with exaggerated fear during the initial phase of the COVID-19 pandemic and reduces the risk of experiencing anxiety and stress symptoms. Females and younger participants were relatively vulnerable to developing these symptoms. We discussed these findings considering some recent studies that reported similar relationships and made recommendations for future research.

## Introduction

The psychological repercussions of the coronavirus (COVID-19) outbreak in the general population are reported by empirical research studies as well as a few systematic reviews of studies that validate the devastating social and psychological implications (Asdaq et al., 2020; Salari et al., 2020; Serafini et al., 2020). Various studies confirmed fear reactions and pervasive anxiety in people due to the sudden high-level global prevalence of disease and uncontrolled impact of the COVID-19 pandemic on various domains of life (Alkhamees et al., 2020; Bäuerle et al., 2020; Wang et al., 2020). The fear reactions were also fueled by the massive media coverage of the impacts of COVID-19 at global, regional, and local levels and lots of misinformation on social media (Gabarron et al., 2021). During the initial phases of the COVID-19 infection, it rapidly influenced many regions of the world (World Health Organization, 2020). Despite some progress in the testing and development of vaccines, World Health Organization (WHO) and public health experts emphasize compliance with social distancing to control its spread. In the Kingdom of Saudi Arabia, and in many other countries, congregational processions and prays were restricted during the peak times of pandemic to abide by social distancing measures implemented by the government (Baunez et al., 2020; Yezli and Khan, 2020). The adherence to social distancing and quarantining of whole cities during the early period of the pandemic resulted in elevated feelings of loneliness and a negative impact on the subjective wellbeing of people (Alkhamees et al., 2020; Rubin and Wessely, 2020; Xiong et al., 2020).

Previous literature suggests that the social, cultural, and religious contexts of individuals and communities play a significant role in determining psychological responses to stressful experiences which impact at the mass level (Ungar, 2013; Blanc et al., 2016). In the wake of the COVID-19 pandemic, public mental health experts advocated for multidisciplinary research priorities to understand and address the mental health consequences of the pandemic at the individual, familial, and community levels (Holmes et al., 2020). Understanding different aspects of people’s spiritual beliefs and religious practices during the COVID-19 pandemic and its relationship with their health and wellbeing has become an important topic of debate and research as the fear propagated during the pandemic (Fardin, 2020). Findings of a study from the United Arab Emirates (UAE) that explored religious coping during the COVID-19 pandemic in Muslim and Christian communities found an inverse relationship between positive religious coping and depressive symptoms especially in Muslim communities this relationship was stronger in comparison to Christians (Thomas and Barbato, 2020). Besides, the study reported a negative relationship between positive religious coping and a history of psychological disorders. There is also an ongoing debate that whether people’s inclination to seek support from spiritual and faith connections during the crisis times is a natural temporary response or it really helps to deal with long-term mental health consequences of crisis. For instance, a study from Poland reported an increase in religious practices among Polish people during lockdown times and the researchers viewed it as a temporary response to the pandemic situation despite that shifts toward religious practices caused by COVID-19 appear to be a real phenomenon and researchers underpinned the need for more research on different aspects of this phenomenon (Boguszewski et al., 2020). A study assessed the relationship of religious coping with mental health in a sample of American Orthodox Jews and findings that an intrinsic religiosity plays a significant role in reducing stress levels and has a positive impact, whereas negative religious coping was inversely related to mental wellbeing (Pirutinsky et al., 2020). A systematic review based on studies from the pre-pandemic period concluded that religiosity and spirituality predict a significant but moderate reduction in the symptoms of clinical depression (Braam and Koenig, 2019) and warranted more investigation in this area. A review of studies conducted during the COVID-19 pandemic from Muslim communities indicated the protective role of spirituality in provision of mental relief during emergency periods and crisis times such as pandemic (Fardin, 2020).

Over the last two decades, an increasing emphasis is demonstrated in the psychological and sociological literature on healing through spirituality/religiosity in both research and clinical practice to promote community health and wellbeing (Hackney and Sanders, 2003; Pargament et al., 2005; Unterrainer et al., 2014). Spiritual practices were found to enhance resilience and positive coping among individuals and may act as protective factors to weaken the psychological effects of such catastrophes (Bryant-Davis and Wong, 2013). The role of religious/spiritual practices and beliefs in the context of stressful and traumatic experiences at a personal level was discussed in previous research (Pargament et al., 1998). The way people frame the crisis event in the context of their religious beliefs may help them to gain a deeper understanding of life, connection with God, and hope (Villas Boas, 2020). Literature reports on the positive relationship between religiosity and psychological health, enhanced self-esteem, and reduced stress levels (Bryant-Davis and Wong, 2013). Recent research illustrated that during the crisis periods such as the COVID-19 pandemic people found solace by connecting or reconnecting with their spiritual beliefs, faith, and religious practices (Boguszewski et al., 2020; Coppola et al., 2021; Rababa et al., 2021). The psychological literature conceptualizes spiritual coping as making use of cognitive resources to gain and maintain mental peace, meaning, and connection during crisis times (Aten et al., 2014; Ozcan et al., 2021). The research on spirituality assessment has shown that spiritual QoL is neither psychological nor social in nature but a distinctive, important entity in its own right. It need not be theorized or operationalized in terms of “objective” indicators like frequency of religious attendance but is readily accessible through subjective self-reports. The analysis of the factor structure of the SRBP scale on the WHO Quality of life scale demonstrated that spirituality stands distinctively and relatively equally alongside other important QoL domains (O’Connell and Skevington, 2010). However, currently, there is inconclusive research to determine the role of spirituality in dealing with pandemic stressors, particularly the unique features of the COVID-19 pandemic which impacted more than half of the world in the era of most advanced medical sciences and technology.

There is ample evidence that validates the heightened levels of stress and anxiety symptoms were experienced by people in various regions of the world during the COVID-19 outbreak including Saudi Arabia (Asdaq et al., 2020; Bäuerle et al., 2020; Cullen et al., 2020; Saltzman et al., 2020). These excessive feelings of anxiety and stress symptoms are attributed to the bewilderment fueled by the media as well as the actual threat that communities are faced with due to the sudden and pervasive nature of the crisis. To our knowledge, in the context of COVID-19 pandemic stress, there is a rise in debate on various social media and other platforms about the role of spirituality and faith to support communities, however, to date there is limited empirical evidence specifically role of spiritual connections in normalizing stress reactions in Saudi Arabia. The current research attempted a baseline investigation of these aspects in the general population in Saudi Arabia. According to the statistical data by the World Population Review (WPR), the population of Saudi Arabia is approximately 35 million Saudi Nationals and 9 million expatriates from various countries of the world ([Bibr B49][Bibr B49]). All the Saudi Nationals are Muslims by religion and share similar cultural values, however, expatriates who belong to various other religions such as Christians, Jews, Hindus, Buddhists, and Sikhs, and different cultural backgrounds. Saudi Arabia is considered primarily a religious society. Every year, Muslims from all over the world visit the two Holy Mosques to practice pilgrimage. Till November 2020, 10,060 cumulative cases of COVID-19 per 1 million population with 159 cumulative deaths per 1 million population were reported in Saudi Arabia (Worldometer, 2020). During the complete and partial lockdown implemented in Saudi Arabia from Feb 2020 till the end of June 2020, people were completely forbidden to visit Holy Mosques during this time. Post-lockdown, the number of visitors to Holy Mosques is controlled by the governing authorities to comply with the preventive measures of the COVID-19 pandemic (Yezli and Khan, 2020).

The present study aims at assessing the protective role of spiritual connections in lessening the anxiety and stress symptoms in the general population. It was hypothesized that spiritual beliefs would have an inverse relationship with anxiety and stress symptoms experienced by the people during the initial phases of the COVID-19 pandemic in Saudi Arabia. The predictive role of spiritual connections was tested after controlling the effect of demographic variables.

## Materials and Methods

### Participants and Procedure

The total sample comprised 795 participants 415 (52%) male and 308 (48%) female respondents residing in Saudi Arabia during the time of this survey. An online survey was considered the most viable approach to collect data in the context of the social distancing protocol which was strictly implemented in Saudi Arabia during the first 2 weeks of July 2020. The lockdown measures were implemented in the first week of July 2022 but lifted in the 2nd week of July 2022. Between the months of April to May 2020, the mortality rate in Saudi Arabia fluctuated between 0.027 and 0.028 per 100 patients and the average infection rate was around 27,000 cases per day (Alyami et al., 2020).

The weblink of the survey questionnaire was shared through various social media platforms and the university’s online portal. The survey was designed keeping in view the strengths and limitations of an online survey questionnaire (Regmi et al., 2016). Keeping in view that respondents are more likely to fill out the survey using a smartphone and tablets the font size and layout of the questionnaire were adjusted in accordance with the average size of smartphone screens used by people in Saudi Arabia which lie between 5 and 5.6 inches. Self-report psychological measures are considered appropriate methods to collect data about people’s thoughts and mental states and the study questionnaire included relevant subscales from standardized psychological measures to collect data for this study. The online questionnaire was comprised of a four page questionnaire including the first page which included a study description and informed consent. All participants agreed on informed consent before completing the survey questionnaire.

### Study Variables and Instruments

#### Background Information

Participants were inquired to provide information about their demographic background including gender, age, nationality, education, marital status, and exposure to the COVID-19 infection.

#### Anxiety and Stress Symptoms

The Anxiety and Stress sub-scales on the shorter Arabic version of DASS-21, assessed the symptoms and levels of anxiety and stress symptoms in participants. Each subscale contains seven items rated on a four-point Likert scale ranging from 0 (did not apply to me at all) to 3 (applied to me very much). The total scores are obtained by the sum of the score on the sub-scales measuring anxiety and stress symptoms. The score obtained is multiplied by two in accordance with the scoring instructors. This continuous data is used to run the statistical analysis to calculate mean differences in stress and anxiety scores across demographic variables and predict the linear relationship between predictors and outcome variables. The internal consistency as calculated on this data is acceptable with Cronbach’s Alpha coefficient for the Stress Scale (α = 0.89), and for Anxiety, (α = 0.81).

#### Use of Spiritual Connections to Cope

The use of spiritual connections to cope during the COVID-19 pandemic was assessed with items on the WHOQOL SRBP WHOQOL spirituality, religiousness, and personal beliefs (?SRPB). The section on spirituality comprises items that focus on assessing the strength of spiritual beliefs and participants were asked to respond to these items keeping in view the context of the COVID-19 pandemic. A sample item is *“To what extent does a connection to a spiritual being help you to get through hard times?”* The response categories (1 = Not at all, 2 = A Little, 3 = A Moderate Amount, 4 = Very Much, 5 = An Extreme Amount). Higher scores indicate more use of spiritual connections to cope during hard times. The internal reliability was found to be (α = 0.89).

### Data Analysis

The data were analyzed by using IBM SPSS software (25.0 version). The descriptive characteristics of the variables under study were described using percentages, mean scores, and standard deviation (S.D.). The data was checked for meeting normality assumptions. *T*-test and ANOVA were applied to see the significance of mean differences in stress, anxiety, and spiritual connections across demographic variables. In a preliminary analysis, stepwise regression models were tested to identify the best model fit. A multiple linear regression analysis was applied to determine the predictive role of the use of spiritual connection in coping with hard times during the pandemic after adjusting the effect of gender and age. In the final analysis, a multiple linear regression analysis was applied to determine the independent predictive relationship of spiritual connection in reducing stress and anxiety symptoms after adjusting for the effect of gender and age. The *p*-value significance was chosen at *p* < 0.05.

### Ethical Approval

The Ethical Committee of the University of Hail authorized the ethical approval of the study which complies with the Declaration of Helsinki. Informed consent was obtained, and the study followed the key ethical principles of beneficence and non-maleficence during all stages of research.

## Results

### Descriptive Characteristics of Sample and Mean Differences Across Background Variables

The sample includes 52% male and 48% female participants with a mean age of 36 years (SD ± 10.9). It comprises 75% Saudi nationals and 25% were from other nationalities. Over half of the participants had had college-level education and around two-thirds were married.

The significance of means differences in anxiety, stress, and spiritual connections are presented in Table 1. Female participants had significantly higher scores on the anxiety, stress, and spiritual connections scales as compared to males (*p* = 0.000). The mean differences were statistically significant across age and across marital status, for anxiety and stress symptoms and these were non-significant on spiritual connections. The mean differences were statistically non-significant across education categories. Saudi nationals had significantly higher scores on the anxiety scale as compared to other nationalities and mean differences were statistically non-significant on the stress scale and coping with spiritual connections.

**TABLE 1:** Percentage values, mean scores, and significance of mean differences across background variables (*n* = 795).

	Variables	Mean (S.D.)	Mean (S.D.)	Mean (S.D.)
	
	*f (%)*	Anxiety	Stress	Spiritual connections
**Gender**	Male (*f* = 415; 52%)	5.74 (3.77)	11.68 (5.26)	13.81 (1.89)
	Female (*f* = 380; 48%)	7.74 (4.16)	13.91 (5.62)	15.48 (1.93)
	*t*-test	*t*(793) = 3.54; *p* < 0.001	*t*(793) = 2.88; *p* < 0.01	*t*(793) = 6.15; *p* < 0.001
	Cohen’s d^(a)^	0.50	0.40	1.04
**Nationality**	Saudi (*f* = 600; 75*%)*	7.08 (4.15)	13.12 (5.59)	14.16 (1.86)
	Non-Saudi (*f* = 195; 25%)	5.55 (3.42)	11.62 (5.01)	14.74 (1.98)
	*t*-test	*t*(793) = 2.62; *p* < 0.01	*t*(793) = 1.68(ns)	*t*(793) = 1.79(ns)
	Cohen’s d	0.40	0.28	0.30
**Age categories**	19–29 years (*f* = 230; 29%)	9.48 (4.51)	16.24 (5.73)	14.41 (1.95)
	30–39 years (*f* = 280; 35%)	6.22 (3.73)	12.48 (5.23)	14.32 (2.04)
	40 years and above (*f* = 285; 36%)	4.88 (3.48)	10.18 (5.07)	15.02 (1.85)
	*F*-test	*F*(2,794) = 22.69, *p* < 0.001	*F*(2,794) = 20.66, *p* < 0.001	*F*(2,794) = 2.68(ns)
	Partial Eta squared^(b)^	0.05	0.05	0.02
**Education**	School level (*f* = 106; 13%)	6.18 (3.99)	11.22 (5.60)	14.18 (1.96)
	College level (*f* = 422; 53%)	6.81 (4.11)	12.09 (5.52)	14.62 (1.95)
	University level (*f* = 267; 37%)	6.74 (3.81)	13.08 (5.30)	14.48 (1.95)
	*F*-test	*F*(2,792) = 0.25(ns)	*F*(2,792) = 1.22(ns)	*F*(2,792) = 1.22(ns)
	Partial Eta squared	0.05	0.05	0.02
**Marital status**	Not married (*f* = 270; 34%)	9.02 (4.42)	15.48 (5.90)	14.44 (2.07)
	Currently Married (*f* = 525; 66%)	5.55 (3.62)	11.34 (5.08)	14.68 (1.89)
	*t*-test	*t*(793) = 5.63; *p* < 0.001	*t*(793) = 4.88; *p* < 0.001	*t*(793) = 0.78(ns)
	Cohen’s d	0.80	0.75	0.12

*^(a)^ Cohen’s d = Small effect size = 0.2; Medium effect size = 0.5; Large effect size = 0.8. ^(b)^ Partial Eta squared values = Small effect size = 0.01; Medium effect size = 0.06; Large effect size = 0.14 or higher. ns = non-significant.*

Table 2 shows that there were statistically non-significant mean differences in anxiety scores, stress scores, and spiritual connections scores between those not exposed to the COVID-19 infection, directly exposed to the COVID-19 infection, and indirectly exposed to the COVID-19 infection in this study sample.

**TABLE 2:** Analysis of variance (ANOVA) to determine significance of difference between those having direct and indirect exposure to the COVID-19 infection (*n* = 795).

Exposure to the COVID-19 infection	Mean (S.D.)	Mean (S.D.)	Mean (S.D.)

*f (%)*	Anxiety	Stress	Spiritual connections
Not exposure (*f* = 570; 72%)	6.72 (4.04)	12.42 (5.58)	14.71 (1.98)
^(a)^Indirect exposure (*f* = 172; 21%)	6.62 (4.04)	13.41 (5.26)	14.4 (1.86)
^(b)^Direct exposure (*f* = 53; 7%)	6.18 (3.31)	12.06 (4.71)	13.81 (1.89)
	*F*(2,792) = 0.11(ns)	*F*(2,792) = 0.61(ns)	*F*(2,792) = 1.39(ns)
^(c)^Partial Eta squared	0.05	0.05	0.02

*^(a)^ Indirectly exposed: Family/friend member living with person got COVID-19 infection. ^(b)^ Person himself/herself diagnosed with COVID-19 infection; ns = non-significant. ^(c)^ Partial Eta squared values = Small effect size = 0.01; Medium effect size = 0.06; Large effect size = 0.14 or higher.*

### Relationship Between Coping With Spiritual Connections and Psychological Symptoms

Table 3 shows results from the multiple linear regression model where anxiety was taken as a dependent variable. The use of spiritual connections to cope significantly reduces the anxiety symptoms by 0.27 units. Being female increases the vulnerability to experiencing anxiety symptoms by 0.88 units. The younger age participants were more likely to experience anxiety symptoms by 0.75 units. The overall model has an adjusted *R*^2^ = 0.074, which means that 7% of the variance in anxiety symptoms is explained by this model. Female respondents were at increased risk to experience anxiety due to the COVID-19 pandemic and spiritual connections despite the small value of beta coefficients in this model demonstrating a statistically significant negative relation with anxiety.

**TABLE 3:** Multiple linear regression model: anxiety as dependent variable (*n* = 795).

	Unstandardized coefficients		Standardized coefficients		
Predictor variables	β	SE	β	*t* (*P*-value)	95% confidence interval
**Constant**	7.61	0.71		10.85	6.23 – 8.99
Using spiritual connections to cope	−0.27	0.07	−0.13	−3.81***	−0.41 – −0.13
Gender	0.88	0.28	0.11	3.07**	0.32 – 1.45
Age in years	−0.75	0.01	−0.019	−5.36***	−0.10 – −0.04
F Change = 21.09; *p* = 0.000 *R*^2^Adj = 0.074					

P-value significance; ***p < 0.001; **p < 0.01; *p < 0.05.

Table 4 shows results from the multiple linear regression model where stress was taken as a dependent variable. Gender, age, and spiritual connections were the significant predictors in the multiple regression model. Use of spiritual connections to cope significantly reduce the stress symptoms by 0.36 units. Being female increases the risk for stress symptoms by 0.92 units. The young age respondents were 0.11 units at increased vulnerability to experience stress symptoms. The overall model has an adjusted *R*^2^ = 0.072, which means that 7% of the variance in stress symptoms is explained by this model. The analysis confirmed the role of spiritual connections in alleviating stress symptoms despite of small beta values it has a statistically significant negative relationship with stress symptoms.

**TABLE 4:** Multiple linear regression model: stress as dependent variable (*n* = 795).

	Unstandardized coefficients		Standardized coefficients		
Predictor variables	β	SE	β	*t* (*P*-value)	95% confidence interval
**Constant**	12.48	0.95		13.01	10.59 – 14.36
Using spiritual connections to cope	−0.36	0.09	−0.13	−3.69***	−0.55 – −0.17
Gender	0.92	0.39	0.85	2.33*	0.14 – 1.70
Age in years	−0.11	0.01	−0.21	−5.67***	−0.14 – −0.71
F Change = 20.53; *p* = 0.000 *R*^2^Adj = 0.072					

P-value significance; ***p < 0.001; **p < 0.01; *p < 0.05.

## Discussion

The present study was conducted to assess the relationship of spiritual connections with psychological symptoms experienced by people during the initial phases of the COVID-19 pandemic in Saudi Arabia. During this time stress and fears related to the COVID-19 outbreak were at peak levels locally and globally. The study findings demonstrated that spiritual connections can be considered a resource to cope during crisis times and are found to be negatively associated with the likelihood of anxiety and stress symptoms. The study is a useful contribution to filling the existing gaps in research in this area because most of the initial studies from Saudi Arabia focused on assessing mental health symptoms in the general population (Alghamdi et al., 2020; Alkhamees et al., 2020), with negligible focus on the contribution of spirituality and religiosity as a resource to cope with mental health symptoms experienced by people. The findings of this study have significant implications which demonstrated the role of spiritual connections in coping during difficult times, and we discussed below these findings in light of the literature on spirituality and mental health and the possible implications of these findings.

In the literature, spirituality has been seen as a source of resilience to deal with stressful situations. It involves cognitive processes where individuals use their spiritual beliefs and sense of inner calmness to cope in difficult times (Braam and Koenig, 2019). Spiritual connections may lead to adaptive coping by influencing the stress appraisal processes. This is also somewhat supported by our study findings where individuals who employed spiritual connections to cope were significantly at lesser risk to experience stress and anxiety. The use of spiritual strength decreases the likelihood of experiencing anxiety and stress symptoms after adjusting for gender and age. These findings align with a recent study from Italy, which showed that spirituality during the pandemic period appeared to play a protective factor with psychological symptoms (Coppola et al., 2021). The protective role of spirituality is primarily due to its specific elements which include a sense of belonging, meaningfulness, and pragmatic interconnections experienced by human beings through spiritual practices (Unterrainer et al., 2014). These factors are responsible for enhancing emotional wellbeing and mental health in individuals, particularly during crisis periods (Pirutinsky et al., 2020). Besides spiritual practices embed the mindfulness elements which help people to deal with apprehensions, nervousness and other anxiety symptoms experienced by individuals due to the COVID-19 pandemic (Timbers and Hollenberger, 2022).

Spirituality focuses on the existential being thus changes the individual’s whole perspective about life challenges and death (Thompson, 2007). The strength of spiritual beliefs may lessen the negative interpretation of the crisis situations and enable people to make use of spiritual connections and to stay calm in uncertain situations (Ozcan et al., 2021). Study findings points toward the contributory role of spiritual beliefs through a negative relationship of spiritual connections with stress and anxiety symptoms. The literature considers spiritual beliefs to serve as the central point of reference point during stressful times (Krause et al., 2016). For instance, in times of communal disasters, engagement in spiritual practices helps people to feel grounded to deal with high levels of fear and uncertainty (Aten et al., 2014).

In our analysis, we adjusted the effect of gender and age because they also appeared as significant predictors. One of the important study findings is the significance of mean differences in anxiety symptoms, stress symptoms, and spiritual connections where female participants had significantly higher scores. The increased reliance on spiritual connections could be due to increased levels of stress and anxiety symptoms experienced by female respondents. These findings are explainable as a recent study also reported the significance of gender differences in stress appraisal, levels of the sense of coherence, and stress coping strategies (Lelek-Kratiuk and Szczygieł, 2022). This study endorsed low levels of sense of coherence in women which increased their risk to experience high levels of stress. Furthermore, they reported that women are more likely to employ a variety of coping strategies in comparison to men. However, women use these coping methods less effectively as reported by another study conducted during the COVID-19 pandemic (Lelek-Kratiuk and Szczygieł, 2022) and this may increase their susceptibility to experiencing stress and anxiety. We also observed in this study that women had significantly higher mean scores on spiritual connections as compared to men and simultaneously scored high on the anxiety and stress scale and this susceptibility is further validated in predictive analysis. Women despite of high scores on spiritual connections were more likely to develop psychological symptoms. The possible explanation for this vulnerability in the light of psychological literature is the intense stress reactivity of women when confronted with stressful situations (Lelek-Kratiuk and Szczygieł, 2022). Besides the actual increase of burden on the women during the COVID-19 pandemic such as care of young children and elders during lockdown along with online work demands may have influenced their sense of coherence and increased the likelihood of experiencing stress and anxiety (Laufer and Shechory Bitton, 2021).

In the context of pandemics, the spiritual beliefs at individual levels as well as religious practices at the individual level have a positive side, however, the collective prayers and religious congregations increased the risk to get the infection. In some Muslim communities, the misinterpretation of religious beliefs and unrealistic ideas about the rapid spread of infection persuaded people to violate precautionary measures during the COVID-19 pandemic. For instance, the belief that *“We are afraid of God only”* or *“All sickness and all health are from God”* or *“Whatever happens to us is God’s will”* are some of the thoughts expressed by people who attended missionary activities in Malaysia (Dein et al., 2020). Another study (Algahtani et al., 2021), examined the religious attitudes and practices of the Saudi community during the COVID-19 pandemic and found the majority of the participants endorsed the suspension of collective prays during the outbreak, however, the participants older than the age of 50 years and young males demonstrated less acceptance toward these bans in comparison to other groups. In our study, we found that increase in age was negatively associated with the experience of psychological symptoms. These findings suggest that the reduced risk of older participants experiencing psychological symptoms during the COVID-19 pandemic in Saudi Arabia could be due to their strong ties with spirituality. The former study also illustrated that older individuals showed more resistance to the banning of collective prayers. This could be due to their strong connections with religious and spiritual sources. However, there are other possible explanations such as one study attributed low adherence with precautionary measure during the COVID-19 pandemic in older people could be due to the low levels of understanding of COVID-19 (Sun et al., 2020). The low levels of anxiety and stress symptoms in older people could be due to poor appraisal of the repercussions due to the COVID-19 pandemic. Thus, coping during the COVID-19 pandemic was determined by a myriad of social, economic, cultural, and religious factors which need to be understood in more depth. Despite, few studies attempted to assess the role of faith in coping during the COVID-19 pandemic in non-Muslim (Timbers and Hollenberger, 2022), and Muslim communities (Bisri, 2020), currently there are many gaps that are recommended to be appropriately addressed in the future research priorities.

While our study provides some evidence about the statistically significant negative relationship between anxiety and stress symptoms with spiritual connections, however, the beta values are small and only 7% of the variance is explained by the regression models. There is a need to validate this relationship in other communities by using other measures of spirituality. Additionally, as reported in the literature people may have turned to seeking comfort through spiritual connections (Coppola et al., 2021) or the widespread fear of the COVID-19 pandemic at the global level could be one possible factor. There is a study from Jordan that reported high levels of death anxiety and low levels of religious coping and spiritual wellbeing experienced by adults (Rababa et al., 2021). However, qualitative data can help us understand the complex nature of the relationship between spiritual experiences, religious practices, and mental health.

In Saudi Arabia, online tools and mobile applications were developed and used to support the execution of religious activities during the COVID-19 pandemic. For instance, the use of the Tawakkalna App regularized the timings and number of attendees during prayers in Holy Mosques over the last 2 years (Hassounah et al., 2020). How such measures supported or hindered the religious activities and spiritual experiences of Muslims has not been assessed to date. Additionally, mosques are though open for adult visitors, but restrictions are still applicable for the past 2 years children are bound to stay outside the boundary walls of the Holy Mosques. This situation impacts the access of mothers with young children to visit or pray. Currently, there is limited data about the religious and spiritual experiences of people from diverse social and religious backgrounds. This is also one of the limitations of this study, which also collected data primarily from Muslim communities living in Saudi Arabia, and the information from people belonging to other religious communities was not gathered. Among the study’s strengths is the large sample size recruited from various regions of Saudi Arabia and the use of standardized scales to assess coping with spirituality and psychological symptoms. However, the limitations of self-report methods need to be considered while interpreting the study findings.

In this study, we presented evidence of the role of spirituality in the relief of stress and anxiety symptoms experienced by people during the pandemic. Some of the research implications are to explore what phenomena in spirituality are more helpful for people to deal with stress reactions when facing chaotic situations. There is certainly a need for longitudinal research studies to clarify the complex nature of the relationship between spirituality and religiosity in mental health. Although the mental health benefits of spirituality and religion have been reported in prior literature (Weber and Pargament, 2014) during normal times, the context of the COVID-19 pandemic was unique in many aspects. The chaos and feelings of helplessness were massive due to their negative impacts at the global level. In many regions of the world, people interpreted this event as punishment from God or supreme power and that could be a source of aggravating the symptoms of anxiety or stress. It would be worth investigating to study the moderating role of spirituality between religious beliefs and mental health symptoms in the context of crisis events. The interaction between spirituality, religion, and mental health needs to be investigated considering the multidimensional nature of these constructs and the environmental context across various regions and cultures.

Some of the practical implications based on our study findings that have demonstrated a link between spirituality and mental health are using spirituality as a tool to cope with anxiety and stress symptoms during uncertain situations such as pandemics. This suggestion is in-line with some other recent literature as mental health experts are re-exploring the significance of spiritual strengths in mental wellness (Cook, 2020; Coppola et al., 2021). For instance, mindfulness and meditation exercises have been found useful to experience inner peace and calmness during stressful times. Spiritual connections provide a sense of coherence to individuals and literature supports those having stronger levels with sense of coherence and more adaptive to adversities (Barni et al., 2020). Findings have shown public health significance and justify the need for mental health screening and community interventions along with strengthening spiritual resources to normalize the psychological symptoms during the outbreaks such as the COVID-19 pandemic. These measures will prevent the mental health repercussions of the pandemics in vulnerable populations and reduce the suffering of communities.

## Conclusion

In conclusion, our study findings demonstrated a significant role of spiritual connections in reducing anxiety and stress levels during the peak period of the COVID-19 pandemic. Older adults were at decreased risk to experience these symptoms and a possible explanation is their strength of spiritual connections whereas this risk is not lessened in the case of women. Factors such as women’s stress reactivity and less effective use of coping strategies may be possible reasons for their increased susceptibility. Besides the use of spiritual connection could possibly be a source of temporary comfort rather than actual relief in this segment of the population. These findings underscore the need for more research to understand various factors during the pandemic that aggravate the mental health sufferings of people with varying religious identities, spiritual practices, and beliefs. The study reinforces the promotion of mental health through the enrichment of various personal and community resources including spirituality.

## Data Availability Statement

The raw data supporting the conclusions of this article will be made available by the authors, without undue reservation.

## Ethics Statement

The studies involving human participants were reviewed and approved by the study was conducted in accordance with the Declaration of Helsinki, and approved by the Institutional Review Board (or Ethics Committee) of University of Hai’l (protocol code 25518//5/42). The patients/participants provided their written informed consent to participate in this study.

## Author Contributions

FA and BA: conceptualization. SH, FA, and BA: methodology. RF, SH, AAh, and SO: data collection. SH and AAl: software. BA, FA, AAh, and IG: validation. RK, FA, RF, and AAh: formal analysis. SH, AAl, and SO: investigation. FA, SO, RF, and AAl: resources. AAh, RK, AAl, and SO: data curation. SH, FA, and BA: writing—original draft preparation. IG, AAl, and RK: writing—review and editing. AAh, IG, and SO: visualization. SH, FA, and AAl: project supervision. FA, AAl, and RF: project administration. RF, SH, AAh, AAl, ST, and RK: funding acquisition. SH and IG: critical intellectual contribution during revision. All authors contributed to the article and approved the submitted version.

## Conflict of Interest

The authors declare that the research was conducted in the absence of any commercial or financial relationships that could be construed as a potential conflict of interest.

## Publisher’s Note

All claims expressed in this article are solely those of the authors and do not necessarily represent those of their affiliated organizations, or those of the publisher, the editors and the reviewers. Any product that may be evaluated in this article, or claim that may be made by its manufacturer, is not guaranteed or endorsed by the publisher.
